# How Patients Take Malaria Treatment: A Systematic Review of the Literature on Adherence to Antimalarial Drugs

**DOI:** 10.1371/journal.pone.0084555

**Published:** 2014-01-20

**Authors:** Katia Bruxvoort, Catherine Goodman, S. Patrick Kachur, David Schellenberg

**Affiliations:** 1 London School of Hygiene and Tropical Medicine, London, United Kingdom; 2 Ifakara Health Institute, Dar es Salaam, Tanzania; 3 United States Centers for Disease Control and Prevention, Atlanta, Georgia, United States of America; Universitat Rovira i Virgili, Spain

## Abstract

**Background:**

High levels of patient adherence to antimalarial treatment are important in ensuring drug effectiveness. To achieve this goal, it is important to understand levels of patient adherence, and the range of study designs and methodological challenges involved in measuring adherence and interpreting results. Since antimalarial adherence was reviewed in 2004, there has been a major expansion in the use of artemisinin-based combination therapies (ACTs) in the public sector, as well as initiatives to make them more widely accessible through community health workers and private retailers. These changes and the large number of recent adherence studies raise the need for an updated review on this topic.

**Objective:**

We conducted a systematic review of studies reporting quantitative results on patient adherence to antimalarials obtained for treatment.

**Results:**

The 55 studies identified reported extensive variation in patient adherence to antimalarials, with many studies reporting very high adherence (90–100%) and others finding adherence of less than 50%. We identified five overarching approaches to assessing adherence based on the definition of adherence and the methods used to measure it. Overall, there was no clear pattern in adherence results by approach. However, adherence tended to be higher among studies where informed consent was collected at the time of obtaining the drug, where patient consultations were directly observed by research staff, and where a diagnostic test was obtained.

**Conclusion:**

Variations in reported adherence may reflect factors related to patient characteristics and the nature of their consultation with the provider, as well as methodological variations such as interaction between the research team and patients before and during the treatment. Future studies can benefit from an awareness of the impact of study procedures on adherence outcomes, and the identification of improved measurement methods less dependent on self-report.

## Introduction

While considerable progress has been made in the last decade to reduce malaria morbidity and mortality, malaria continues to cause more than 200 million cases and more than 600,000 deaths per year [1]. The vast majority of deaths occur among children under five in Africa, though many other parts of the world are also affected. Malaria is entirely preventable and treatable, but if treatment is delayed or ineffective, the parasite burden may rapidly increase and cause severe malaria, which has a case fatality rate of 10–20% even among those receiving treatment [2]. Resistance of parasites to antimalarials, exacerbated by their widespread and indiscriminate use, threatens the effectiveness of malaria treatment.

In order for antimalarial treatment to be effective, multiple steps must occur [3]–[4]. The patient must promptly seek care, the correct diagnosis must be made; the correct drug and dose must be recommended; the drug must be efficacious, of good quality and in stock; the patient must receive or purchase the correct dose; and the correct dose must be taken with correct timing until all doses are complete. Not only can incomplete dosage result in treatment failure, but it may arguably contribute to the spread of resistance [5]–[6]. Sub-therapeutic treatment can result in recrudescence and select for resistant parasites [7]. Patient adherence, defined as correctly taking the full therapeutic course of treatment, is thus a critical step in ensuring antimalarial effectiveness and reducing malaria mortality.

To achieve this goal, it is important for policymakers to understand levels of patient adherence to antimalarials, how they vary by context, and how adherence can be improved. However, studies measuring patient adherence encounter substantial methodological challenges, such as selection of appropriate definitions of adherence and appropriate measurement methods. This results in a broad diversity of study designs which, along with the wide range of study contexts and different antimalarial drugs, can challenge interpretation of adherence results.

The use of antimalarial drugs was last reviewed by Yeung and White in 2004 [8]. Of the 22 studies they identified in Africa, Asia and South America that reported quantitative data on patient adherence, only five assessed adherence to artemisinin-based combination therapies (ACTs), and only eight studies, mostly household surveys, measured adherence to antimalarials obtained through community health workers or drug retailers. Since publication of this review, there has been a major expansion of the availability of ACTs, which have been shown to be efficacious and may reduce the spread of resistance in low transmission settings [9]–[12]. Due to the development of resistance to older antimalarials, such as chloroquine and sulfadoxine pyrimethamine (SP), ACTs have become the first-line treatment for *Plasmodium falciparum* malaria in the public sector in most malaria-endemic countries. In addition, a growing number of initiatives to increase ACT use through community health workers and private sector providers have been implemented [13]. Furthermore, a large number of new studies assessing adherence to antimalarials, particularly to ACTs, have been conducted in the last nine years, raising the need for an update on this topic.

Here, previously reviewed and recent studies providing quantitative results on adherence to antimalarials obtained for treatment are analysed. We examine how results vary by definition of adherence and key methodological characteristics, and we present the studies' own findings on factors associated with adherence. We emphasize challenges in measuring adherence, avoiding bias, and implications for future research.

## Methods

Studies included in this review were identified by three methods. First, a systematic literature search was conducted on PubMed using MeSH and free text terms as follows: (Medication Adherence (MeSH) or Patient Compliance (MeSH) or compliance or adhere*) and (Antimalarials (MeSH) or antimalarial*). Secondly, reference lists from studies and reviews identified were searched manually for relevant studies. Finally, researchers known to be currently active in the field were contacted.

Studies that were clearly irrelevant were immediately discarded, and abstracts and manuscripts of the remaining studies were examined in detail to determine relevance. Published studies that provided quantitative data on patient adherence to antimalarials obtained for treatment of malaria were included in this review. Where papers employed both quantitative and qualitative methods, only the quantitative results are reported here. Studies were included from all parts of the world in any language utilizing various study designs, including household surveys and clinical trials examining the effectiveness of supervised versus unsupervised treatment that specifically reported data on adherence in the unsupervised arm. Studies assessing adherence to antimalarials obtained for prophylaxis, and effectiveness studies that did not report data on adherence were excluded. Manuscripts of studies meeting inclusion criteria were read in detail and data on study settings, objectives, study design, definitions of adherence, methods of assessing adherence and results were systematically reviewed and abstracted into a database.

## Results

**Figure 1:**
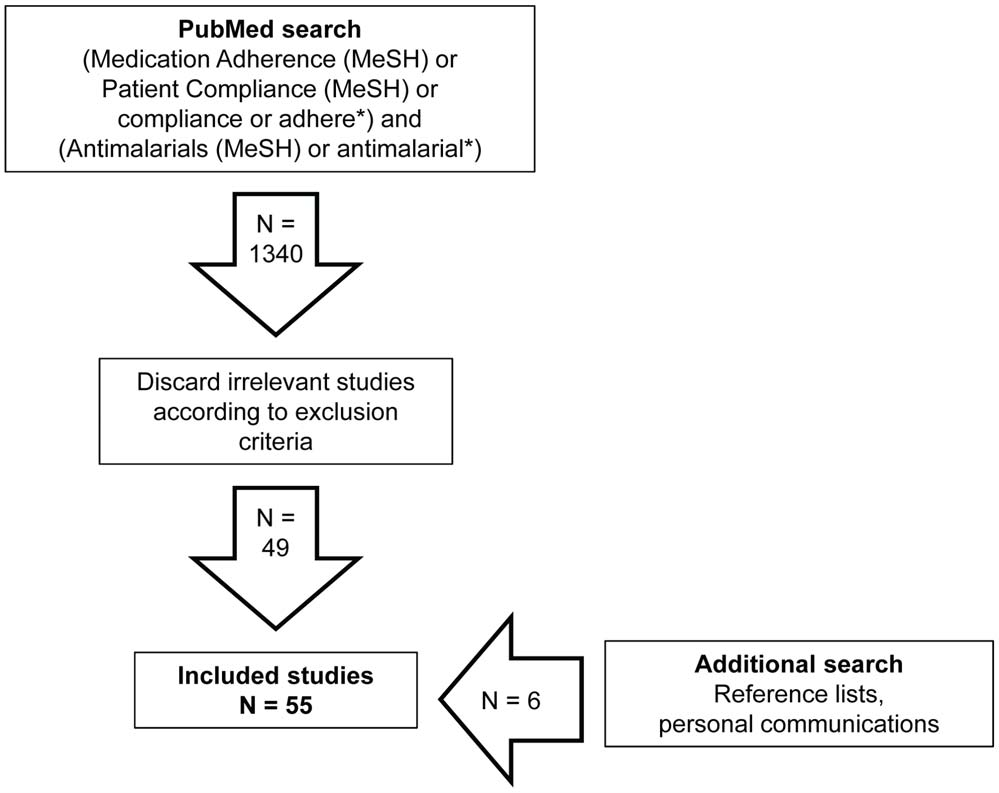
Literature search results.

The initial literature search using PubMed identified 1340 studies (Figure 1). In total, 49 studies were retained from the initial search. Many of the excluded studies referred to antimalarials obtained for prophylaxis or treatment of conditions other than malaria. Manual examination of reference lists and personal communication with other researchers in the field identified six additional studies, making a total of 55 studies.

### Characteristics of studies included

Three main types of studies were identified: descriptive studies, interventions to improve adherence, and studies with clinical outcomes as a primary endpoint (Tables 1–3). While there is clearly some overlap between types, studies were categorised as descriptive except for those that described an intervention to improve adherence or simultaneously measured clinical outcomes and patient adherence. Distinguishing studies with clinical outcomes is helpful, as they were often conducted under relatively controlled conditions, or with relatively intensive follow-up, which may have influenced adherence results.

**Table 1:** Characteristics of studies included in the review (by author) for descriptive studies.

Study, (Country), Source(s) of drugs	Drug regimen(s)^1^	Method(s) of assessing adherence	Approach(es) to assessing adherence^2^	Day of follow-up visit (Day 1 = drug dispensed)	Level of adherence (N = denominator)
Abuaku et al. 2004 [35], (Ghana), Multiple sources	SP, chloroquine (CQ), & amodiaquine (AQ)	Household survey questionnaire	Completed treatment	n/a	SP - 100% (N = 4); CQ - 11.1% site 1 (N = 171); CQ - 36.4% site 2 (N = 195); AQ - 12.9% site 1 (N = 9); AQ - 50% site 2 (N = 2)
Ajayi et al. 2008 [36], (Ghana, Nigeria, Uganda), Community health workers	AL in Nigeria and Uganda & artesunate-amodiaquine in Ghana	Household survey questionnaire	Completed treatment	n/a	Ghana - 97% (N = 153); Nigeria - 93% (N = 60); Uganda - 81% (N = 31); Overall - 94% (N = 244)
Amin et al. 2004 [37], (Kenya)	SP & amodiaquine	Household survey questionnaire	*Unique approach* = patients who took a higher dose than recommended or an adequate dose	n/a	SP - 66.7% (N = 78); AQ - 13.8% (N = 94)
Barnes et al. 2005 [38], (South Africa), Health facilities	AL	Household survey questionnaire	Completed treatment	n/a	96% (N = 235)
Beer et al. 2009 [14], (Zanzibar), Health facilities	Artesunate-amodiaquine (3 days)	Self-report, pill count	Verified completed treatment & *Unique approach* = Verified completed treatment, plus child did not vomit dose, or another dose was administered if child vomited first dose	Day 4	Verified completed treatment - 77% (N = 174); *Unique approach* - 63% (N = 174)
Chinbuah et al. 2006 [15], (Ghana), Community health workers	AL (3 days)	Self report	Timely completion	Day 4	100% (N = 334)
Cohen et al. 2012 [16], (Uganda), Private drug shop	AL (3 days)	Self-report, pill count	Verified completed treatment	Day 4	65.8% (N = 152)
Deming et al. 1989 [39], (Togo), Multiple sources	Chloroquine	Household survey questionnaire	Completed treatment	n/a	29% (N = 370)
Depoortere et al. 2004 [17], (Zambia), Health facility (refugee)	SP + artesunate (3 days)	Self-report, pill count	Verified timely completion	Day 4	39.4% (N = 162)
Depoortere et al. 2004 [18], (So. Sudan), Health facility	AL (3 days)	Self-report, pill count	Verified timely completion	Day 4	59.1% (N = 93)
Fogg et al. 2004 [19], (Uganda), Health facilities	AL (3 days)	Self-report, pill count, lumefantrine assay^3^	Verified timely completion	Day 4	90% (N = 210)
Gerstl et al. 2010 [20], (Sierra Leone), Health facilities	Artesunate-amodiaquine (3 days)	Self-report, pill count	Verified timely completion	Day 4	48% (N = 118)
Kabanywanyi et al. 2010 [22], (Tanzania), Health facility	AL (3 days)	Self-report	Timely completion & Completed treatment	Randomized to follow-up visit close to time of one of the five doses to be taken at home	Timely completion - 90% (N = 552); Completed treatment - 98% (N = 552)
Kachur et al. 2004 [23], (Tanzania), Health facility	SP + artesunate (3 days)	Self-report, pill count	Timely completion & Verified timely completion	After 48 hours	Timely completion - 76.6% (N = 128); Verified timely completion - 75% (N = 128)
Kalyango et al. 2013 [24], (Uganda), Community health workers	AL (3 days)	Self-report, pill count	Verified completed treatment & *Unique approach* = took as prescribed with fatty meals at each dose and no vomiting within 30 minutes	Day 4	Verified completed treatment - 86% (N = 667); *Unique approach* - 16.9% (N = 667)
Khantikul et al. 2009 [25], (Thailand), Health facilities	Chloroquine + primaquine (14 days)	Self-report	Completed treatment	Up to one year	24.8% (N = 206)
Kolaczinski et al. 2006 [26], (Uganda), Health facilities	Chloroquine + SP (3 days)	Self-report, pill count	Verified completed treatment	Day 4	96.3% (N = 241)
Krause & Sauerborn 2000 [4], (Burkina Faso), Multiple sources	Antimalarial drugs (mostly chloroquine & quinine)	Pill count	*Unique approach* = drugs taken correctly according to count of pills in the middle of the treatment course	Middle of the treatment course	68% (N = 47)
Lawford et al. 2011 [27], (Kenya), Health facilities	AL (3 days)	Self-report, pill count	Verified completed treatment	Day 4	64.1% (N = 918)
Lemma et al. 2011 [28], (Ethiopia), Community health workers	AL (3 days)	Self-report, pill count	Verified timely completion & Completed treatment	Day 4	Verified timely completion - 38.7% (N = 155); Completed treatment - 73.5% (N = 155)
Mace et al. 2011 [29], (Malawi), Health facilities	AL (3 days)	Self-report, pill count	Verified timely completion & Verified completed treatment	Day 4	Verified timely completion - 65% (N = 386); Verified completed treatment - 75% (N = 386)
Nshakira et al. 2002 [30], (Uganda), Multiple sources	Chloroquine (3 days)	Self-report	Completed treatment	Day 4	37.8% (N = 463)
Nsungwa-Sabiiti et al. 2005 [40], (Uganda), Multiple sources	Chloroquine & chloroquine + SP	Household survey questionnaire	Completed treatment	n/a	25% (N = 65)
Onyango et al. 2012 [41], (Kenya), Multiple sources	AL	Household survey questionnaire	Completed treatment	n/a	47% (N = 297)
Peeters Grietens et al. 2010 [21], (Peru), Health facilities	Primaquine (7 days)	Self-report, triangulation with health centre records	Completed treatment & *Unique approach* = self-reported adherence plus health centre records verifying that patients returned to receive the last four doses of primaquine	Up to one year	Completed treatment - 71.9% (N = 185); *Unique approach* - 62.2% (N = 185)
Pereira et al. 2011 [31], (Brazil), Health facilities	Chloroquine + primaquine (7 days)	Self-report, pill count	Verified timely completion	Day 7	86.4% (N = 280)
Reilley et al. 2002 [32], (Sri Lanka), Health facility	Chloroquine + primaquine (5 days)	Self-report	Completed treatment	Day 6	74% (N = 132)
Simba et al. 2012 [33], (Tanzania), Health facilities	AL (3 days)	Self-report, lumefantrine assay^3^	Timely completion	Day 7	88.3% (N = 444)
Thera et al. 2000 [42], (Mali), Multiple sources	Chloroquine	Household survey questionnaire	Completed treatment	n/a	37.8% (N = 152)
Twagirumukiza et al. 2010 [34], (Rwanda), Health facility	Quinine tablets (7 days, last 4 unsupervised)	Self-report, pill count, electronic pill boxes	Verified timely completion & *Unique approach* = percentage of doses taken according to electronic pill box	Day 8	Verified timely completion - 100% (N = 56); *Unique approach* - 82.7% (N = 56)

^1^ Duration of drug regimen in days not given for household surveys;

^2^ See Table 4 for definitions of approaches;

^3^ Not incorporated into approach to assessing adherence.

**Table 2:** Characteristics of studies included in the review (by author) for studies assessing interventions to improve adherence.

Study, (Country), Source(s) of drugs	Drug regimen(s)^1^	Intervention	Method(s) of assessing adherence	Approach(es) to assessing adherence^2^	Day of follow-up visit (Day 1 = drug dispensed)	Level of adherence without intervention (N = denominator)	Level of adherence with intervention (N = denominator)
Agyepong et al. 2002 [43], (Ghana), Health facility	Chloroquine (3 days)	Drug labels & verbal instructions	Self-report	Timely completion & *Unique approach* = at least the minimum dose or higher taken once per day	Day 4	Timely completion^4^ - 24% (N = 205); *Unique approach* - 45% (N = 205)	Timely completion (control) - 27% (N = 78); Timely completion (intervention) - 39% (N = 121); *Unique approach* (control) - 44% (N = 78); *Unique approach* (intervention) - 78% (N = 121)
Ansah et al. 2001 [44], (Ghana), Health facility	Chloroquine (3 days)	Introduction of tablets to replace syrup	Self-report, pill count or measurement of remaining syrup^3^	Timely completion	Day 4	42% (N = 144)	91% (n = 155)
Denis et al. 1998 [45], (Cambodia), Multiple sources	Quinine + tetracycline (7 days)	Posters & video	Self-report, pill count^3^	Completed treatment	Day 7 (Day 4 if doses were purchased for only 3 days)	Group 1 - 1% (N = 95); Group 2 - 10% (N = 82)	Group 1 (posters and video) - 39% (N = 88); Group 2 (posters only) - 15% (N = 120)
Kangwana et al. 2011 [46], (Kenya), Multiple sources	AL (3 days)	Subsidised AL, shopkeeper training, & community awareness activities	Self-report	Completed treatment	n/a	Group 1 - 40.5% (N = 26); Group 2 - 53.1% (N = 30)	Group 1 (control) - 24.8% (N = 89); Group 2 (intervention) - 67% (N = 221)
Lauwo et al. 2006 [47], (Papua New Guinea), Health facility	Chloroquine + SP (3 days)	Packaging & counselling	Self-report	Timely completion	Day 4	76.5% (N = 119)	Counselling - 92.9% (N = 112); Counselling & packaging - 95.5% (N = 91)
Marsh et al. 1999 [48], (Kenya), Private drug shops	Chloroquine	Shopkeeper training	Household survey questionnaire; laboratory assay in a subset of children given a full dose^3^	Completed treatment	Day 4	3.7% (N = 109)	75% (N = 108)
Marsh et al. 2004 [49], (Kenya), Private drug shops	Chloroquine & SP	Shopkeeper training	Household survey questionnaire	Completed treatment	n/a	Chloroquine - 8% (N = 160)	SP - 64% (N = 441)
Okonkwo et al. 2001 [50], (Nigeria), Health facilities	Chloroquine	Pictorial insert & verbal instructions	Self-report, measurement of remaining syrup	Verified timely completion	48 hours after start of treatment	36.5% (N = 190)	Pictorial insert - 51.9% (N = 225); Pictorial insert & verbal instructions - 73.3% (N = 217)
Qingjun et al. 1998 [51], (China), Health facilities	Chloroquine + primaquine (8 days)	Packaging	Self-report, laboratory assay	Timely completion & Biological assay (by phenobarbital markers)	Day 4 or Day 9	Timely completion - 83% (N = 163); Biological assay - 80.5% (N = 134)	Timely completion - 97% (N = 161); Biological assay - 97% (N = 138)
Shwe et al. 1998 [52], (Myanmar), Health facilities	Artesunate + mefloquine (3 days)	Packaging & training	Laboratory assay	Biological assay (by chloroquine & quinine markers)	Day 7	n/a	99.5% (N = 380)
Sirima et al. 2003 [53], (Burkina Faso), Community health workers	Chloroquine	Packaging & availability through community health workers	Household survey questionnaire	Completed treatment	n/a	n/a	52% (N = 1806)
Winch et al. 2003 [54], (Mali), Community health workers	Chloroquine (3 days)	Community health worker training	Self-report, pill count or measurement of remaining syrup	Timely completion & *Unique approach* = Timely completion or higher dose than recommended	Day 4	Timely completion - 1.5% (N = 131); *Unique approach* - 21.6% (N = 131)	Timely completion - 42.1% (N = 151); *Unique approach* - 71.7% (N = 151)
Yeboah-Antwi et al. 2001 [55], (Ghana), Health facilities	Chloroquine (3 days)	Age-based packaging of syrup & tablets	Self-report, pill count or measurement of remaining syrup^3^	Timely completion	Day 4	Tablets - 60.5% (N = 152); Syrup - 32.6% (N = 95); Overall - 49.8% (N = 247)	Tablets - 82.0% (N = 167); Syrup - 54.7% (N = 95); Overall - 72.1% (N = 262)

^1^ Duration of drug regimen in days not given for household surveys;

^2^ See Table 4 for definitions;

^3^ Not incorporated into adherence definition;

^4^ Weighted results for three control and three intervention clinics.

**Table 3:** Characteristics of studies included in the review (by author) for studies with clinical outcomes which also report adherence.

Study, (Country), Source(s) of drugs	Drug regimen(s)^1^	Approach(es) to assessing adherence^2^	Approach(es) to assessing adherence^2^	Day of follow-up visit (Day 1 = drug dispensed)	Level of adherence with intervention (N = denominator)
Achan et al. 2009 [56], (Uganda), Health facility	AL (3 days) & quinine (7 days)	Self-report, pill count	*Unique approach* = percentage of pills taken	Day 4	AL - 94.5% (N = 85); Quinine - 85.4% (N = 75)
Bell et al. 2009 [57], (Malawi), Health facility	AL (3 days) & chloroproguanil-dapsone (CPD, 3 days)	Self-report, electronic pill boxes, laboratory assays^3^	Completed treatment & *Unique approach* = electronic pill bottle opened once on Day 1 & two times each on Days 2 & 3	Day 8	Completed treatment (AL) - 100% (N = 185); Completed treatment (CPD) - 99.2% (N = 371); *Unique approach* (AL) - 92% (N = 87); *Unique approach* (CPD) - 90.6% (N = 181)
Congpuong et al. 2010 [63], (Thailand), Health facilities	Artesunate + mefloquine + primaquine (3 days)	Self-report, drug assays	Completed treatment & Biological assay	Day 4	Completed treatment - 100% (N = 240); Biological assay (mefloquine marker) - 96.3% (N = 215); Biological assay (quinine marker) - 98.5% (N = 214)
Duarte et al. 2003 [67], (Brazil), Health facilities	Quinine + doxycycline (7 days) & primaquine + chloroquine (14 days)	Self-report	Completed treatment	Up to 4 months	83.8% (N = 488)
Dunyo et al. 2010 [58], (The Gambia), Health facilities	AL (3 days) & chloroproguanil-dapsone (CPD, 3 days)	Self-report (pill count for some^3^)	Completed treatment	Day 4	AL - 67% (N = 600); CPD - 94% (N = 599)
Faucher et al. 2009 [60], (Benin), Health facility	AL (3 days) & artesunate-amodiaquine (ASAQ) (3 days)	Self-report, pill count	Verified completed treatment	Day 4	AL - 83% (N = 96); ASAQ - 91% (N = 96)
Fungladda et al. 1998 [59], (Thailand), Health facility	Artesunate (4 days) & quinine + tetracycline (7 days)	Self-report, pill count	Verified completed treatment	Day 5 or Day 8	Artesunate - 98.4% (N = 61); Quinine + tetracycline - 71.7% (N = 53)
Na-Bangchang et al. 1997 [64], (Thailand), Health facilities	Artemether + mefloquine (2 days)	Laboratory assays	Biological assay	Day 3	86.8% (N = 106)
Rahman et al. 2008 [61], (Bangladesh), Health facility	AL (3 days)	Self-report, pill count, lumefantrine assay^3^	Verified timely completion	Day 4	93% (N = 160)
Souares et al. 2008 [65], (Senegal), Health facilities	SP + amodiaquine (3 days)	Self-report, laboratory assays^3^	Timely completion & *Unique approach* = at least 80% of the prescribed dose of each of the two drugs was taken	Day 4	Timely completion - 37.7% (N = 289); *Unique approach* - 64.7% (N = 289)
Takeuchi et al. 2010 [62], (Thailand), Health facility	Chloroquine + primaquine (14 days)	Self-report	Completed treatment	Day 8 & Day 15	85% (N = 101)
Yepez et al. 2000 [66], (Ecuador), Health facilities	Chloroquine + primaquine (3 days for *Pf* & 7 days for *Pv*)	Self-report	Timely completion	Day 4 or Day 8	*Pf* - 79.2% (N = 120); *Pv* - 58.5% (N = 129); Overall - 68.3% (N = 249)

^1^ Duration of drug regimen in days not given for household surveys;

^2^ See Table 4 for definitions;

^3^ Not incorporated into adherence definition

More than half of the 55 studies were descriptive (30 studies) [4], [14]–[42]. The majority of these (21 studies) were observational follow-up studies [14]–[34], where patients obtaining a drug were visited at their home or returned to the drug outlet after a specified number of days, at which time adherence data were collected. While most follow-up studies were prospective, two studies retrospectively identified patients to follow-up for adherence assessments [21], [25]. Several of these studies were part of larger studies that included an intervention (e.g. use of community health workers [15], [24] or subsidization of ACTs in private retail outlets [16]), but did not provide information on the impact on adherence through pre and post or control group comparisons, so the studies were categorised as “descriptive” in terms of their assessment of adherence. Eight studies used household surveys to collect descriptive data [35]–[42], and one study used both household survey and follow-up methods [4]. In these household surveys, households in selected areas were visited without prior knowledge of who had obtained antimalarial drugs, and interviews were conducted about episodes of illness occurring in the weeks prior to the survey, treatment obtained, and adherence.

Thirteen studies evaluated interventions to improve adherence [43]–[55]. Of these, seven were randomized controlled trials (RCTs) [44], [46]–[47], [50]–[51], [54]–[55], two were controlled pre- and post-intervention studies [43], [45], two were uncontrolled pre- and post-intervention studies [48]–[49], and two were post-intervention only adherence assessments [52]–[53]. Follow-up methods were used by eight of the thirteen intervention studies, while the remaining four used household surveys. The interventions included new packaging with and without training, including pre-packaging of two component drugs together and pictorial inserts to packaging [47], [50]–[53], [55], as well as dispenser training of shopkeepers [48]–[49] or community health workers [54]. Ansah *et al.* (2001) [44] conducted an RCT of chloroquine tablets for children compared to chloroquine syrup, while Denis *et al.* (1998) [45] evaluated videos and posters as community health education strategies to improve adherence to a 7-day regimen of quinine + tetracycline.

The third type of studies, those assessing clinical outcomes as a primary endpoint in addition to reporting patient adherence, included seven RCTs comparing effectiveness and adherence of different drug regimens [56]–[60] or supervised versus non-supervised treatment [61]–[62], and four uncontrolled studies also assessing effectiveness and adherence [63]–[66], all of which employed follow-up methods. In addition, a prospective open cohort study examined the association of previous compliance with antimalarials for malaria caused by *P. falciparum* or *P. vivax* and occurrence of malaria during follow-up [67].

Of the 55 studies, 40 took place in Africa, 11 in Asia, and four in Latin America. Subjects included all age groups in 25 studies, only children under five in 19 studies, both children under five and older children in an additional seven studies, and only adults in four studies. Most studies assessed adherence to antimalarials taken to treat infection with *P. falciparum*, with five studies focusing on treatment for *P. vivax* [21], [25], [31], [51], [62], and three studies on treatment for both species [32], [66]–[67]. Most studies assessed adherence to treatment obtained in health facilities or malaria clinics. Four follow-up studies evaluated adherence to drugs obtained from community agents [15], [24], [28], [54] three took place in the context of complex humanitarian emergencies [17]–[18], [26], and three were conducted from private drug shops [16], [30], [45]. Most household surveys reported adherence to antimalarials obtained from both public and private sectors, except for four that focused on interventions to improve adherence to antimalarials obtained from drug shops [48]–[49] or community health workers [36], [53].

Patient adherence to more than one drug regimen was assessed in 12 studies, while 43 studies reported adherence to a single drug (Tables 1–3). Adherence to ACTs was assessed in 26 studies. Artemether-lumefantrine (AL) was the ACT in 18 of these studies, with two of these 18 also reporting adherence to artesunate-amodiaquine [36], [60]. Other ACTs evaluated included two additional studies of artesunate-amodiaquine [14], [20], as well as SP + artesunate [17], [23] and artesunate + mefloquine [52], [63]–[64]. Non-artemisinin-based combinations featured in 13 studies (chloroproguanil-dapsone (CPD) [57]–[58], quinine + doxycycline or tetracycline [45], [59], [67], chloroquine + SP [26], [40], [47], SP + amodiaquine [65] and, for treatment of *P. vivax* malaria, chloroquine + primaquine [25], [31]–[32], [51], [62], [66]–[67]). Adherence to chloroquine and other monotherapies was assessed in 20 studies.

### Definitions of adherence and measurement methods

The 55 studies reviewed here employed a wide range of definitions and methodologies. Adherence was measured by questionnaires containing varying detail about how and when drugs were taken (self-report); physical counts of tablets remaining in packaging or dispensing envelopes (pill counts) and volumetric measurement of syrups; pill containers with electronic caps that record the date and time of each opening (electronic pill boxes); assays for drug levels in biological samples; and composites of these methods.

At least one approach used in 52 of the 55 studies could be classified under one of five overarching approaches defined for the purpose of this review, based on both the nature of adherence required and the method used to measure adherence (Table 4). “Completed treatment” identifies individuals who said they completed treatment. “Verified completed treatment” refers to reported completed treatment that is corroborated by a pill count. “Timely completion” refers to patients reporting that they completed each dose at an appropriate time. “Verified timely completion” identifies those reporting timely completion with a pill count to confirm that no tablets were left. Lastly, “biological assay” refers to detection of sufficient levels of drugs in biological samples.

**Table 4:** Approaches to assessing patient adherence across studies.

Approach	Definition	Method	Number of studies^1^
Completed treatment	Patient completed treatment	Self-report	28
Verified completed treatment	Patient completed treatment	Self-report and pill count	10
Timely completion	Patient exactly followed instructions in terms of dose, frequency and duration	Self-report	12
Verified timely completion	Patient exactly followed instructions in terms of dose, frequency and duration	Self-report and pill count	11
Biological assays	Sufficient levels of drug(s) in biological samples	Biological assays	4
Unique approaches	Various	Various	11

^1^ All studies are included if adherence is reported by at least one of these five approaches (n = 52 studies) and are included more than once if multiple approaches were used.

Correct timing of doses, involving the correct dose, frequency, and duration, was required in 22 studies (“timely completion” and “verified timely completion”), 11 of which were studies of ACTs. However, there was considerable variation in which intervals were considered “correct”, “recommended” or “prescribed”. Several studies calculated the expected time of each dose per the manufacturer's instructions and allowed an interval of several hours on either side [22]–[23], [28], while other studies required the correct dose to be taken on each day specified, or for AL twice per day for three days [15], [18], [33], [61], and other studies did not report exactly what was considered correct. This is in contrast to assessments of “completed treatment” and “verified completed treatment”, which did not require correct timing of doses. Furthermore, many studies reported in their methods that drug packaging was inspected, but only 21 studies specifically incorporated pill counts into adherence definitions, requiring self-reported adherence verified by empty packages or the expected number of remaining pills (“verified completed treatment” and “verified timely completion”).

### Adherence results

The studies reported a very wide range of results for the percentage of patients adherent, ranging from 1.5% to 100% across different studies and settings. Below we explore how the results varied firstly by the approach to assessing adherence and data collection, secondly by antimalarial and outlet type, and thirdly by the nature of the interaction between patients and dispensers or researchers during the study. Scatter plots are used to facilitate the identification of general patterns in these results. Finally we present the studies' own findings on factors found to be associated with adherence in multivariate models.

i. Variation by approach and data collection method

**Figure 2:**
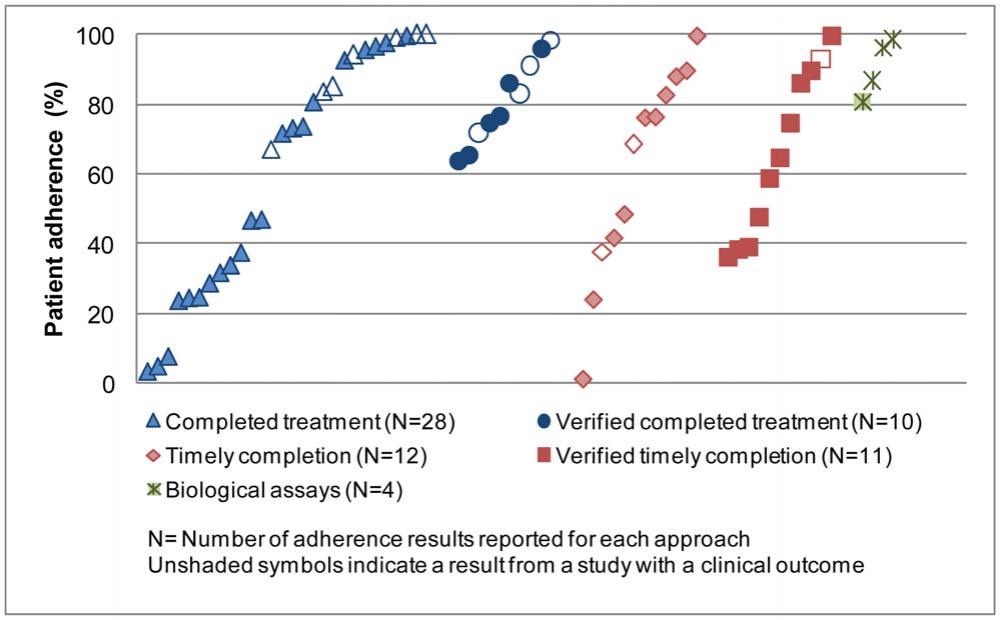
Percentage of patients classified as adherent, by Approach to assessing adherence.

Figure 2 shows a comparison of adherence results by the five approaches. The plot includes multiple points from studies which used more than one approach to report adherence. Studies that did not use any of the five approaches were not plotted [4], [37], [56]. In addition, when results of adherence to the same drug were reported from more than one study site within the same country, the weighted average of these sites was plotted [35], [45]. For intervention studies, only baseline results were plotted in order to represent standard practice; thus, two studies were not plotted since they provided adherence results post-intervention only [52]–[53]. When multiple non-overlapping degrees of adherence were used (such as *definitely non-adherent, probably non-adherent, probably adherent*), the most adherent level was considered the proportion adherent for the purpose of Figure 2.

Overall, it does not appear that using stricter approaches involving correct dose timing (“timely completion” and “verified timely completion”) or requiring pill counts in addition to self-reported histories (“verified completed treatment” and “verified timely completion”) are associated with lower adherence, but this does not account for differences in contexts and methodologies. However, among studies of AL, adherence by “verified timely completion” (38.7%–65%) [18], [28]–[29] was lower compared to “timely completion” (88.3%–100%) [15], [22], [33], except in studies where the research team enrolled patients at the time the drug was obtained and likely had a more significant research presence than in other studies (90% and 93%) [19], [61]. Similarly, adherence to AL by “verified completed treatment” (64.1%–83%) [16], [24], [27], [29], [60] tended to be lower than for “completed treatment” (67%–100%) [22], [28], [36], [38], [57]–[58], with the exception of two household surveys without pill counts with adherence of 47% [41], [46].

Household surveys, which all used the “completed treatment” approach and assessed adherence from both public and private community sources, tended to have lower adherence results than studies with other designs, particularly studies with primary clinical outcomes (Tables 1–3). In addition, studies plotted before implementation of an intervention had lower adherence for all approaches, as is particularly evident in the community-based interventions by Marsh *et al*. (1999, 2004) and Winch *et al.* (2003) and the private-sector follow-up study by Denis *et al.* (2008); this may be because most of the interventions included in the review are older studies and the interventions (e.g. pre-packaging of drugs) have become a standard part of antimalarial treatment used in the newer studies.

Among studies using unique approaches, two studies used electronic pill boxes (Medication Events Monitoring Systems – MEMS™) to measure adherence [34], [57]. In the study by Bell *et al.* (2009) adherence by self-report (“completed treatment”) was 100% for AL and 99.2% for CPD, but by the electronic pill boxes, adherence was 92% for AL and 91% for CPD. Similarly, in the study by Twagirumukiza *et al.* (2010), adherence to quinine tablets was 100% by both self-report (“verified timely completion”) and pill count (no pill boxes had pills remaining), but only 78% of patients took at least 80% of the doses based on the electronic pill box data [34].

Results using biological assays to assess adherence were high (above 90%), but this accounted for only a few studies [15], [51], [64]. Qingjun *et al.* (1998) evaluated a packaging intervention to improve adherence to chloroquine + primaquine marked with phenobarbital to detect concentrations in plasma, while Na-Bangchang *et al.* (1997) measured adherence to artesunate + mefloquine by whole blood mefloquine concentrations based on a reference interval [64]. Similarly, Congpuong *et al.* (2010) used both whole blood mefloquine concentrations and plasma concentrations of primaquine [63] to detect adherence to artemether + mefloquine + primaquine. One additional study (Shwe *et al.*, 1998) also found high adherence of 99.5%, but was not included in the plots because adherence to artesunate + mefloquine was only reported after implementation of a co-packaging and training intervention; in this study, tablets of quinine and chloroquine were added to the regimen as markers for detection by urine assays. Five other studies measured plasma levels of lumefantrine using HPLC with mass spectrometry or UV detection [19], [33], [57], [60]–[61], but adherence was not reported on the basis of these assays. Median lumefantrine concentrations were not significantly different between patients who were or were not considered adherent by self-report (“completed treatment” and “timely completion”) or self-report with pill count (”verified timely completion”).

ii. Variation by antimalarial type and outlet type

The pattern of adherence results between antimalarials was not clear. Across all approaches and by “completed treatment” adherence to AL (47%–100%) [22], [28], [36], [38], [41], [46], [57]–[58] was higher than both adherence to monotherapies estimated from household surveys (3.7%–34%) [35], [39]–[40], [42], [48]–[49] and adherence to longer primaquine regimens for the treatment of vivax malaria (25%–85%) [21], [25], [31]–[32], [51], [62], [66]–[67]. Adherence to AL by “verified completed treatment” (64.1%–83%) [16], [24], [27], [29], [60] was lower than adherence to artesunate-amodiaquine (77%–91%) [14], [60] and chloroquine+SP (96%) [26]. However, adherence to AL by “timely completion” was high in three studies (88.3%–100%) [15], [22], [33] in contrast with studies of SP + amodiaquine (37.7%) [65] and SP + artesunate (76.6%) [23]. By “verified timely completion” adherence to AL was similar in three studies (38.7%–65%) [18], [28]–[29] to adherence to other ACTs (39.4%–75%) [17], [20], [23] and higher in two other studies (90%–93%) [19], [61].

Although most studies evaluated adherence to antimalarials obtained in the public sector, the two descriptive private sector follow-up studies had low adherence, with Nshakira *et al.* (2002) reporting adherence of 37.8% to chloroquine by “completed treatment”, and Cohen *et al.* (2012) describing adherence of 65.8% to AL. Three household surveys [46], [48]–[49] and one follow-up study [45] assessing interventions in private drug stores and surrounding communities also all reported adherence of less than 50%. Adherence where antimalarials were obtained from CHWs in four studies using follow-up methods ranged widely from 1.5%–100% [15], [24], [28], [54], with a study of AL by Lemma *et al.*(2011) in Ethiopia finding adherence of 38.7% by “verified timely completion” and 73.5% by “completed treatment”. In addition, a study evaluating adherence to ACTs dispensed by CHWs reported high adherence of 83%–97% by “completed treatment” in household surveys in three countries [36].

iii. Variation by nature of interaction of patients with dispensers and research personnel

**Figure 3:**
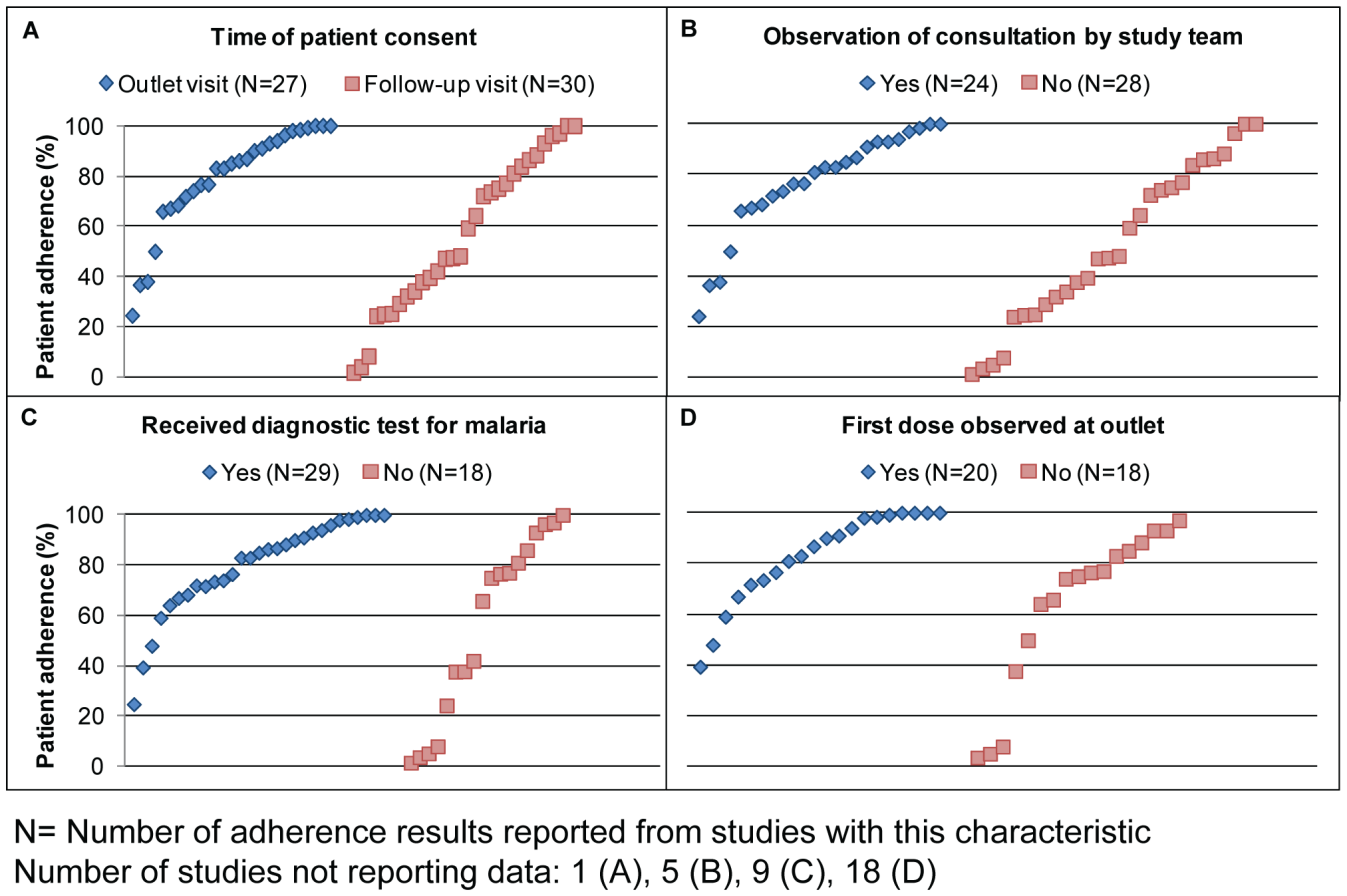
Percentage of patients classified as adherent, by patient interaction with research staff and dispensers.

We explored how adherence results varied depending on the nature of the interaction reported between patients and their dispensers, and between patients and research personnel. Figure 3a–d shows how patient adherence (as assessed by any of the five approaches) varied with four aspects of patient interaction that we hypothesised might influence adherence results. As shown in the first plot, patients in some studies were asked for informed consent to participate in the study at the outlet upon obtaining the drug, while patients in other studies were not asked for informed consent until a later follow-up visit, having had several days to take the drug (Figure 3a). Secondly, research staff in some studies observed the consultation of the patient with the dispenser or conducted the consultation themselves, while other studies did not (Figure 3b). Studies where most patients obtained a malaria diagnostic test prior to treatment were plotted in comparison to studies where patients were not tested (Figure 3c). The fourth plot compares studies where dispensers did and did not observe the patient swallowing the first dose of the drug (Figure 3d). Results of all studies that used one of the five approaches are plotted, as described previously for Figure 2, except that for studies using multiple approaches to assess adherence, only the most inclusive approach reported was plotted (i.e. “completed treatment”). Studies could not be plotted if the nature of the patient interaction for each of the four plots was not reported.

Figure 3a suggests that collecting informed consent from patients at the outlet visit when the drug is dispensed can result in higher adherence compared to requesting informed consent at the time of the follow-up visit. Similarly there is an indication that observation by the study team of patients' consultations with dispensers may influence patients to be more adherent (Figure 3b), and that where patients were confirmed to have malaria with a rapid diagnostic test (RDT) or blood smear prior to being dispensed antimalarials, adherence was higher than among those not tested (Figure 3c). There is also some indication that studies where dispensers observed patients' first dose had higher adherence than those where the first dose was not observed, although the pattern is less clear (Figure 3d).

iv. Factors associated with adherence in multivariate models

Understanding the characteristics and behaviours associated with patient adherence to antimalarials is vital to designing interventions to improve appropriate use of ACTs. Twenty-four studies used multivariate analysis to examine factors associated with adherence: of these, 13 studies reported 30 factors significantly associated with adherence in multivariate models, nine studies found 12 factors associated with non-adherence, and five studies reported not finding any factors significantly associated with adherence or non-adherence [32], [44], [50], [55], [61]. While many of the twenty-four studies tested similar factors, such as demographics, instructions given and patient knowledge, there was substantial diversity in which factors were found significant.

Tables 5–6 show factors significantly associated with adherence (Table 5) and non-adherence (Table 6), including demographics, treatment-seeking behaviour, factors related to the consultation, behaviour, knowledge and perceptions, and satisfaction. Factors significantly associated with adherence in more than one study included higher education [14], [16], [41], higher household income [33], [41], provision of better information on how to take drugs [16], [29], [31], and knowledge about malaria and antimalarials [20], [25], [27]–[28], [66]. Factors significantly associated with non-adherence in more than one study included being male [31], [56], lack of education [17], [19], and vomiting [24], [56]. There were contrasting results for the effects on adherence of patient age and the number of days after onset of symptoms that treatment was sought. Older age of the patient was associated with adherence in one study [27] and non-adherence in another [65], while two other studies found younger age associated with adherence [41] and non-adherence [29]. Similarly, Lemma *et al.* (2011) found that patients who waited more than one day to seek care after onset of fever were more likely to be adherent, but other studies showed that seeking care within 24 hours of symptom onset was associated with adherence [27], and waiting two or more days was associated with non-adherence [24], [62].

**Table 5:** Factors associated with adherence in multivariate models (p<0.05 or 95% confidence interval crosses the null).

Factors	Studies
*Demographics*	
* Education*	
- Caretaker education at least 7 years	Beer et al. 2009 [14]
- Attending some secondary school or beyond	Cohen et al. 2012 [16]
- Higher education	Onyango et al. 2012 [41]
Residence in one of two areas in study location	Duarte et al. 2003 [67]
*Age*	
- Respondent age 25-50 years versus less than 25 years	Lawford et al. 2011 [27]
- Patient age 15 years or more versus less than 15 years	Lawford et al. 2011 [27]
- Patient age less than 13 years	Onyango et al. 2012 [41]
Ownership of radio	Lemma et al. 2011 [28]
Higher household income	Onyango et al. 2012 [41]
	Simba et al. 2012 [33]
*Treatment-seeking behaviour*	
Not having sought treatment at a public health facility	Cohen et al. 2012 [16]
Respondent sought treatment within 24 hrs of symptom onset versus waiting longer	Lawford et al. 2011 [27]
Delay of more than 1 day in seeking treatment after the onset of fever	Lemma et al. 2011 [28]
Previous care sought	Souares et al. 2008 [65]
*Factors related to the consultation*	
Having received exact number of pills to complete treatment	Beer et al. 2009 [14]
Reporting having been given instructions at the shop	Cohen et al. 2012 [16]
Reporting that instructions given were clear	Cohen et al. 2012 [16]
Attended Migowi HC (one of three study outlets)	Mace et al. 2011 [29]
Package used as visual aid by dispenser to explain how to take the drug	Mace et al. 2011 [29]
Received written instructions	Pereira et al. 2011 [31]
Quality of history taking (i.e. nurses at the consultation asked questions about history, symptoms, and previous care)	Souares et al. 2008 [65]
*Behaviour*	
Took first AL dose at HC	Mace et al. 2011 [29]
Taking AL with food or oil	Simba et al. 2012 [33]
*Knowledge and perceptions*	
Knowledge that only mosquitoes cause malaria	Gerstl et al. 2010 [20]
Knowledge of malaria aetiology	Khantikul et al. 2009 [25]
Respondent had seen the drug before	Lawford et al. 2011 [27]
Being able to cite at least one correct instruction on how to take AL	Lawford et al. 2011 [27]
Belief that malaria cannot be treated traditionally	Lemma et al. 2011 [28]
Access to information about antimalarials	Khantikul et al. 2009 [25]
Knowledge of the seriousness of the infection/knowing the species in mixed transmission areas	Yepez et al. 2000 [66]
*Satisfaction*	
Having an improved condition at follow-up	Cohen et al. 2012 [16]
Lower expectation of getting malaria in the next 30 days	Cohen et al. 2012 [16]
Did not report dislikes/side-effects to medication	Lawford et al. 2011 [27]
Preference for AL	Mace et al. 2011 [29]
Satisfaction with received information	Souares et al. 2008 [65]

**Table 6:** Factors associated with non-adherence in multivariate models (p<0.05 or 95% confidence interval crosses the null).

Factors	Studies
*Demographics*	
Being male	Achan et al. 2009 [56]
	Pereira et al. 2011 [31]
Caretaker having different mother tongue to pharmacist	Depoortere et al. 2004 [17]
*Education*	
- Caretaker education (none versus some)	Depoortere et al. 2004 [17]
- Lack of formal education	Fogg et al.2004 [19]
*Age*	
- Being a child under 5	Mace et al. 2011 [29]
- Being a child age 8–10 years versus 2–4 years	Souares et al. 2008 [65]
Head of household profession (retailer/employee vs. farmer)	Souares et al. 2008 [65]
*Treatment-seeking behaviour*	
No fever reported	Kalyango et al. 2013^1^ [24]
Seeking care after 2 or more days	Kalyango et al. 2013^1^ [24]
	Takeuchi et al. 2009^2^ [62]
*Factors related to the consultation*	
Treatment with oral quinine versus AL	Achan et al. 2009 [56]
Being counselled about what to do in case of vomiting	Kachur et al. 2004 [23]
Not understanding instructions	Kalyango et al. 2013^1^ [24]
*Knowledge and perceptions*	
Caregiver's perception that illness is not severe	Kalyango et al. 2013^1^ [24]
*Satisfaction*	
Vomiting	Achan et al. 2009 [56]
	Kalyango et al. 2013^1^ [24]

^1^ Includes patients receiving Al only and AL plus antibiotics (treatment group not significant in multivariate analysis);

^2^ Associated with non-adherence in the second week of primaquine treatment for *P. vivax* infection.

## Discussion

Extensive variation was observed in patient adherence to antimalarials, with many studies reporting very high adherence (90–100%) and others finding clearly suboptimal adherence, sometimes of less than 50%. This may be an important problem, both in terms of clinical outcomes and also in the context of the development of resistance to artemisinin in South-East Asia [68]. However, it is unclear how good adherence must be for ACTs to be efficacious, and which features of adherence (such as correct timing of dose intervals or taking each dose with a fatty meal) matter most.

We identified five overarching approaches to assessing adherence based on recall (“completed treatment” and “timely completion”), recall and pill counts (“verified completed treatment” and “verified timely completion”) and on biological assays. By “completed treatment” and “verified completed treatment”, adherent patients were defined as completing the full course of treatment though not necessarily following a specific schedule. Whether these are appropriate approaches to assess adherence should be considered in light of the pharmacology of the specific drug: if the safety or efficacy of the drug is critically dependent on the timing of the doses then it will be important to assess this when evaluating adherence. As these approaches do not include the spacing of the doses, it is possible for patients to have taken some doses too close together or even to have taken all doses at one time and still be considered “adherent”, though such practices could be of concern for drug safety and efficacy. Furthermore, there is potential variation within each approach in what was considered correct treatment, with some studies taking into account national guidelines on the correct dose-for-weight that the patient should have consumed and other studies assuming the correct amount was obtained.

By “timely completion” and “verified timely completion”, adherent patients were defined as exactly following instructions in terms of dose, frequency and duration according to their responses to interview questions. As noted above, there was considerable variation in definitions of “correct” timing, which may have affected comparability within these approaches. More information is needed on how precise time intervals between doses must be in order for drugs to be efficacious. For example, the packaging of various brands of AL states that the second dose should be taken eight hours after the first dose, which would fall in the middle of the night if the drug is obtained in the evening. In this situation it is unclear whether a patient should still be considered adherent if they take the drugs first thing the next morning instead.

The majority of the adherence studies used one or more of these approaches relying primarily on self-reported drug histories, which may be susceptible to recall and social desirability bias. Studies in Tanzania and Cambodia found high levels of antimalarials circulating in the blood among patients stating they had not taken any drugs in the previous 28 days [69]–[70]. Patients may not accurately recall information about the quantity of drugs taken. Moreover, even if the precise time of obtaining the drug from the provider is known, asking patients when each dose was taken is problematic as they may not have had clocks available or may not know or remember the exact time. Recall bias is likely to be higher in data obtained from household surveys, where interviewers frequently ask about drugs taken in the previous 14 days, compared to follow-up studies, where recall time is usually 4–7 days. Even with short recall periods, patients may not correctly remember details related to each dose. Cultural and demographic factors may also affect the reliability of self-reported data [71]. For example, in a study of the impact of the length of recall periods for health surveys, different recall periods gave different results, and these differences were shown to vary by income group [72].

To avoid being seen as ignorant or negligent, patients who are aware of the expected behaviour may say they were adherent even if they actually were not. A study by Peeters Grietens *et al.* (2010) found that while 72% of patients reported taking the full course of primaquine, only 49% claiming to take the full course had actually received the full course according to records [21]. Likewise, Bell and colleagues stated that self-reported data, which resulted in 100% adherence to AL and CPD in Malawi, was unreliable compared to MEMS™ containers [57].

In order to reduce recall and social desirability bias, some studies incorporated manual examination of drug packaging into their definitions of adherence (“verified completed treatment” and “verified timely completion”). For studies of AL, these approaches yielded lower adherence results than the equivalent approaches without the pill counts (“completed treatment” and “timely completion”). However, even results including pill counts may over-estimate true adherence as removing pills from blister packs does not guarantee that the pills were consumed. Similarly, opening electronic pill boxes does not guarantee a dose was consumed. Patients may have “played” with their pill boxes, opening them without removing pills, or alternatively, they may also have removed multiple doses at one opening, either to discard, consume, or save until the appropriate time.

Despite the limitations of self-reported and pill count approaches, Souares *et al.* (2008) suggested that self-reported data may be more reliable and feasible than assays for drug levels, which require invasive sample collection and complicated field logistics [68]. Drug assays were rarely used for measuring adherence, and their utility and appropriate role remains unclear. Adherence evaluated by the detection of drugs in biological assays was very high (90–100%) in four studies, but these studies assessed adherence to drugs other than AL and involved close interaction of the research staff with patients and in some cases extended follow-up periods. The five studies that reported measuring lumefantrine concentrations, but did not incorporate these assays into adherence results, did not find significant differences in lumefantrine concentrations between patients adherent and non-adherent by self-report. This may have been due to the metabolic variability of the study population, including age, pregnancy, concomitant fat intake and other factors affecting drug absorption, limiting the value of quantitative assessments of patient adherence [73]–[74]. Methods of collecting blood samples, sample preservation under field conditions, and details of the assays themselves are also likely to affect results.

Regardless of the approach used for assessing adherence, Hawthorne bias may occur if a patient's awareness of being studied positively influences medication-taking behaviour. Similarly, if researchers observe patient consultations with the dispenser, this may positively influence the care and advice provided by the dispenser and/or patients' attentiveness and adherence to the treatment. In the studies reviewed here, adherence was higher when informed consent was collected at the time of obtaining the drug and to some degree when patient consultations were directly observed (Figures 3a and 3b). While it is reasonable to assume that medication-taking behaviour of patients who are not aware they are being studied more accurately reflects behaviour in real life contexts, these concerns must be balanced by practical constraints, such as fulfilling other study objectives and the need to obtain the patient's consent and address for follow-up visits.

Some specific patient-dispenser interactions might also be expected to improve adherence. For example, confirmation of diagnosis of malaria by an RDT or blood smear might increase adherence if patients are more aware that they are suffering from malaria, and if patients with confirmed malaria see a better response to treatment than those who have other conditions. Observing the first dose of treatment is another commonly recommended practice and was found to be significantly associated with adherence to AL in one study [29]. We found some indication that malaria diagnosis was associated with higher adherence in the reviewed studies, although the effect was less marked for observing the first dose on adherence overall.

In addition to the approach to measurement and the nature of the patients' consultations, other factors often hypothesised to influence adherence include patient characteristics, antimalarial type and outlet type. However, it was not possible to discern clear patterns across the studies reviewed. There was some evidence from multivariate studies that patients who had higher socio-economic status and were better educated or informed had higher adherence. While there is some concern that the greater number of tablets required for treatment with ACTs (i.e. 24 for an adult) contributes to lower adherence compared to antimalarials requiring fewer tablets, this was not clear in the studies reviewed here. One potential explanation for this is that ACTs often come in co-formulated or co-packaged blister packs, with different coloured packages for each age or weight group. This is in contrast to loose tablets dispensed into paper envelopes, which was often the case for older antimalarials. Not only can the dispenser give the patient the incorrect number of tablets, but the tablets may need to be cut in half to achieve the appropriate doses, and it may be more difficult for the patient to remember how many to take. Secondly, more effective antimalarials such as ACTs may encourage higher patient adherence; if drugs are perceived to be ineffective, patients may use a drug briefly or not at all before looking for a more effective alternative [8]. Finally, perceptions of side-effects may cause variation in adherence across antimalarials, with drugs such as chloroquine and quinine known to have more common minor adverse effects than ACTs such as AL.

It was hard to assess variation across outlet types as of the 55 studies included, only five specifically evaluated adherence to antimalarials from private drug shops [16], [30], [45], [48]–[49] and five from community health workers [15], [24], [28], [36], [54]. However, there were some indications that adherence was relatively low from private sector outlets, highlighting the need for more studies to evaluate adherence to ACTs obtained in this sector and to design interventions to ensure drugs are used appropriately. Interventions to improve adherence that are currently being tested in the private sector include the introduction of RDTs [75]–[76], new packaging, SMS reminders to patients [77], and SMS reminders to drug shop dispensers to encourage them to advise patients on the importance of adherence [78].

## Conclusion

The literature reports extensive variation in patient adherence to antimalarials. The unsatisfactory patient adherence sometimes reported to ACTs obtained in the public sector, and the current dearth of data from the private sector, represent significant challenges for maximising the impact of ACTs. Variations in adherence may reflect factors related to patient characteristics and knowledge, their treatment seeking behaviour, and the nature of their consultation with the provider. However, methodological variations between studies are also likely to be an important source of variability in results, including the methods used for collecting data, and any interaction between the research team and patients before and during the treatment course. Future studies could be strengthened by a greater awareness of the impact of study procedures on adherence outcomes, and the identification of improved measurement methods that are less dependent on self-report.

## Supporting Information

Checklist S1 **PRISMA checklist.**
(DOC)

Flow Diagram S1 **PRISMA flow diagram.**
(DOC)
