# Supplementary material for: How Patients Take Malaria Treatment: A Systematic Review of the Literature on Adherence to Antimalarial Drugs
Source: PLoS One. 2014 Jan 20;9(1):e84555. doi: 10.1371/journal.pone.0084555 (PMC3896377; doi:10.1371/journal.pone.0084555)
Supplement: Flow Diagram S1 — PRISMA flow diagram. (DOC) [file pone.0084555.s002.doc]

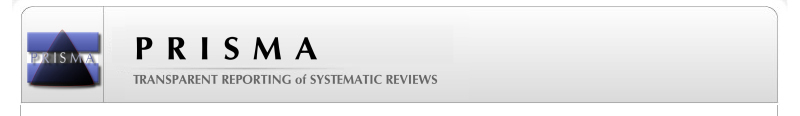
**PRISMA 2009 Flow Diagram**

**Screening**

**Included**

**Eligibility**

**Identification**

Records identified through database searching
(n = 1340)

Additional records identified through other sources
(n = 6 )

Records after duplicates removed
(n = 1346 )

Records screened
(n = 1346 )

Records excluded
(n = 1244 )

Full-text articles assessed for eligibility
(n = 102 )

Full-text articles excluded, with reasons
(n = 47 )

Studies included in qualitative synthesis
(n=55)

Studies included in quantitative synthesis (meta-analysis)
n/a
